# Reinvestigation of the Two-step Synthesis of Sevoflurane

**Published:** 2015

**Authors:** Abolghasem Moghimi, Mostafa Vojdani, Ali R. Banan, Ahmad Mollaei, Mojtaba Mahmoodian, Sayyed Mojtaba Moosavi

**Affiliations:** a *Department of Chemistry, Imam Hossein University, Tehran, Iran. *; b *Department of Nuclear Medicine, The Educational, Research and Clinical Center, Dr. Masih Daneshvari Hospital, Tehran, Iran.*

**Keywords:** Inhalation anesthetic, Sevoflurane, Halogen-exchange fluorination, Hexafluoro-2-propanol

## Abstract

Improvements in the two-step synthesis of 1,1,1,3,3,3-hexafluoro-2- (fluoromehoxy)propane (Sevoflurane) that result in the product cost reduction, safety level enhancement and positive environmental impacts are described. This process consists of chloromethylation reaction of 1,1,1,3,3,3-hexafluoro-2-propanol (HFIP) followed by a halogen-exchange fluorination. This is the first synthesis of Sevoflurane in Iran which was successfully scaled up. During this work, several improvements have been achieved by optimization of the reaction time, the amount of consumed starting materials and solvents and work up procedure while keeping the yield and purity intact. The reaction time of the first step (24 h) was diminished to 4 h. ^19^F NMR spectroscopy was used to investigate the rate of the reaction in the first step and to evaluate the influence of different parameters mentioned on the achieved improvements.

## Introduction

**Scheme 1:**
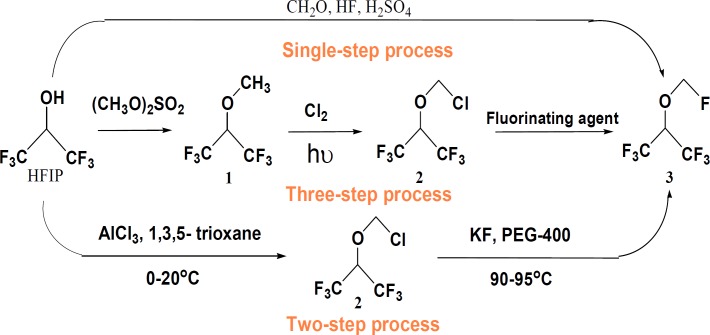
The single, two and three-step synthetic methods of Sevoflurane from hexafluoroisopropanol

Sevoflurane,1,1,1,3,3,3-hexafluoro-2-( fluoromehoxy )-propane, is an important and widely used nonflammable general inhalation anesthetic in the world. Although a number of methods have been introduced by different research groups (1-4), three methods have gained more interest for the industrial production of Sevoflurane (Scheme 1). The single-step synthetic process involves the reaction of 1,1,1,3,3,3-hexafluoro-2-propanol (HFIP) and stoichiometric excess of paraformaldehyde and HF in the presence of fuming sulfuric acid. In this one pot method, a large quantity of chemically aggressive species may cause corrosion of the plant during the process and would also circumvent the problems associated with the handling of highly toxic HF. The three-step method involves, i) reaction of HFIP with a methylating agent such as dimethylsulfate to form sevomethyl ether (1), ii) photochemical chlorination of sevomethyl ether 1 to form Sevochlorane (2), and iii) substitution of the chlorine of Sevochlorane through a Halogen-exchange reaction. In this process, dimethylsulfate and chlorine gas must be handled which are both toxic. In addition, the low yield of the second step was the other disadvantages of this process. 

The third process has been introduced by C. Bieniarz *et al.* as a two-step, efficient, safe and amenable method. The overall yield has been differently reported from 65 to 70% with purity in the range of 99.40-99.95% depending on the procedure (2, 3).

Our investigations on this process, clarified some disadvantages that could be partially or totally removed. The disadvantages are: i) long reaction time, 24 h, for the chloromethylation step and ii) large amount of KF and PEG-400 used in the second step, the fluoride exchange reaction. Herein, some modifications that have been applied on this method are discussed in order to reach a more cost-effective process and to decrease the environmental pollutions, compared with other references (2, 3), with no change in yield and purity of the product (Table 1).

**Table 1:** A comparison with other references

			**Ref 3**	**Ref 2**	**Ref 6**		**Present work**
					**Two step process**	**Single pot** **process**	
**Chloromethylation Step**	**Mixing**	AlCl3 (mol)	0.139 c	64.54	0.139^ c^	32.37	0.26
		HFIP (mol)	0.139 c	64.54	0.139 c	32.37	0.24
		1,3,5-Trioxane (mol)	0.047 c	21.58	0.046 c	10.79	0.08
		Reaction Time (h)	24	24	20 f	Did not mention	4
		HCl gas a	Evaluated	Absorbed d	Evaluated	Absorbed d	Reused i
		Monitoring the reaction	By GC	By GC	By GC	By GC	By 19F NMR
	**Quenching**	HCl 6N (L)	0.050	26.6	0.050 g	13.3	0.040
		Water (L)	Did not mention clearly	10	0.050	5	0.040
		Aqueous Phase b	Did not mention	Siphoned off	Did not mention	Siphoned off	Analyzed by 19F NMR j
		Quenching Time	Did not mention	Did not mention	Did not mention clearly	Did not mention	50 minuets k
**Fluorination Step**	SVC (mol)		0.010	Did not mention e	0.010	Did not mention e	0.18
	KF (mol)		0.040	Did not mention	0.040	97.26	0.22
	PEG-400 (L)		0.010	32	------ h	16	0.050

a Produced during the reaction.

b Produced by washing the mixture.

c The number is rounded. ^d^By scrubbers containing water.

e The SVC didn’t separate because this reaction has been described as a one-vessel process.

f The reaction didn’t completed at this time because the mixture still had a little (<5% quantitatively) amount of bis-HFIP-acetal.

g Cold (-20 °C.).

h diethylene glycol was used as a solvent (0.010 L).

i The HCl gas was directed to a water bath and, after pH adjustment, was used for quenching the chloromethylation step.

j The aqueous phase contains some SVC that was extracted by a solvent.

k The quenching time depends on the reaction scale.

## Experimental

*Chemicals and apparatus*

Hexafluoro-2-propanol, AlCl_3_, 1,3,5-trioxane, KF, and PEG-400 were purchased from Merck and used as received. Sevoflurane was obtained from Abbott. GC analysis was performed using a Varian 3400 gas chromatograph with a flame ionization detector (FID) through a 2m x l/8" OV-101 on 80/100 CWHP packed GC column. ^1^H, ^19^F and ^13^C NMR were recorded on Bruker NMR spectrometers at 250, 235 and 62.5 MHz, respectively. Chemical shifts are reported in ppm downfield from tetramethylsilane (TMS, δ0.00).

*Synthesis of Sevochlorane*

Anhydrous AlCl_3_ (34.9 g, 0.26 mol) was placed into a jacketed glass reactor. The reaction vessel was cooled to 0 ºC, and HFIP (40 g, 0.24 mol) was added in a single portion while stirring. 1,3,5-trioxane (7.2 g, 0.08 mol) was added in portions to the homogeneous slurry of HFIP and AlCl_3_.The generated HCl gas was directed into a water vessel. After 2 h, the temperature of the reaction mixture was increased to 25 ºC. After 4 h, the reaction mixture was cooled to 0 ºC and the careful dropwise addition of cooled water (40 g) started. When adding water was completed, and the exothermic reaction was subsided, 6 N HCl (40 mL) was added rapidly in one portion. Then, the bath temperature was increased to ambient temperature and stirrer rate was increased to dissolve all the remaining aluminate salts. Consequently, three clear layers appeared. The bottom layer (Sevochlorane phase) was separated and washed twice with water and then dried over MgSO_4_ to afford 45 g (87.2%) highly pure Sevochlorane. Data for 1,1,1,3,3,3-hexafluoro-2-(chloromethoxy)-propane (1): bp = 76 °C; ^1^H NMR (250.1 MHz, CDCl_3_): δ 5.57 (s, 2H) 4.54 (septet, 1H, ^3^*J*_HF_ = 5.7 Hz); ^19^F NMR (235.4 MHz, CDCl_3_): δ -74.14 (d, ^3^*J*_HF _= 5.9 Hz ); ^13^C NMR (62.9 MHz, CDCl_3_): δ 121.0 (q, ^1^*J*_FC_ = 283.0 Hz) 80.4 (s), 73.8 (septet, ^2^*J*_FC_= 31.4 Hz).

*Halogen-exchange fluorination*

PEG-400 (50 mL) was placed into a jacketed glass reactor. KF (12.9 g, 0.22 mol) was added thereafter while stirring. Then, Sevochlorane (40 g, 0.18 mol)was added to the mixture and the reaction mixture was heated at 90 ºC for 2 h, and then cooled down to room temperature. Water (50 mL) was added to the mixture. Two clear phases had formed. The bottom phase was separated, dried over MgSO_4_, and distilled to afford 27 g (72%) of highly pure Sevoflurane (99.9%). Data for 1,1,1,3,3,3-hexafluoro-2-(fluoromethoxy)propane (Sevoflurane, 3): bp = 58.5 °C;^ 1^H NMR (250.1 MHz, CDCl_3_): δ 5.42 (d, 2H, ^2^*J*_HF_ = 53.5 Hz) 4.42 (septet, 1H, ^3^*J*_HF_ = 5.7 Hz); ^19^F NMR (235.4 MHz, CDCl_3_): δ -75.13 (dd, ^3^*J*_HF _= 4.7 Hz, ^5^*J*_FF_= 2.4 Hz ), -155.65 (septet of t, ^2^*J*_HF _= 54.13 Hz, ^5^*J*_FF_= 2.4 Hz); ^13^C NMR (62.9 MHz, CDCl_3_): δ 121.1 (q, ^1^*J*_FC_ = 283.0 Hz) 103.1 (d, ^1^*J*_FC_ = 226.4 Hz), 74.24 (septet, ^2^*J*_CF_= 33.4 Hz).

## Results and Discussion

The first modification applied for the two-step process was the first step reaction time that was decreased to 4 h by changing the ratio of the starting materials. The next modification was reducing the amount of solvent and KF, in the second step, with no change on the yield and purity of the product. These modifications would change the original two-step method into a real cost-effective process. 

**Figure 1:**
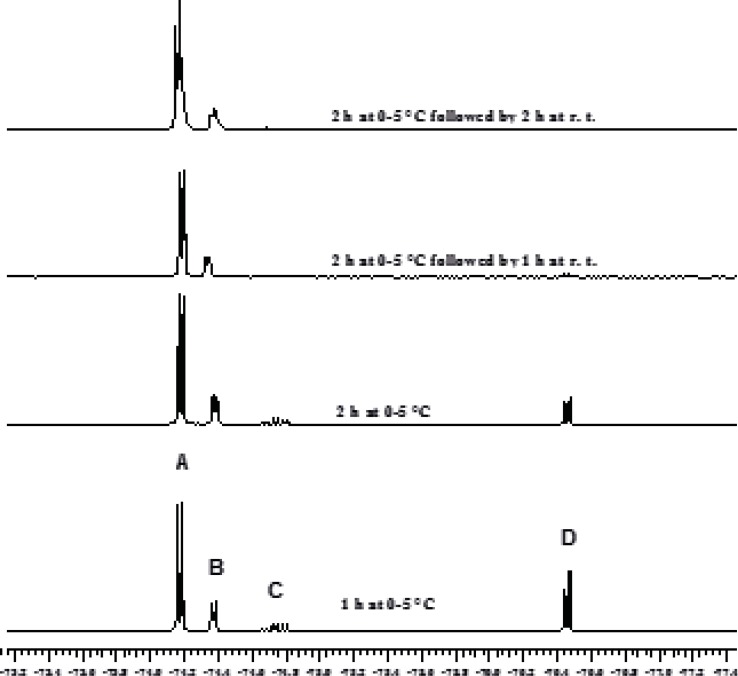
Monitoring the Chloromethylation Reaction by ^19^F NMR Using 10% Excess of AlCl_3_.

**Figure 2:**
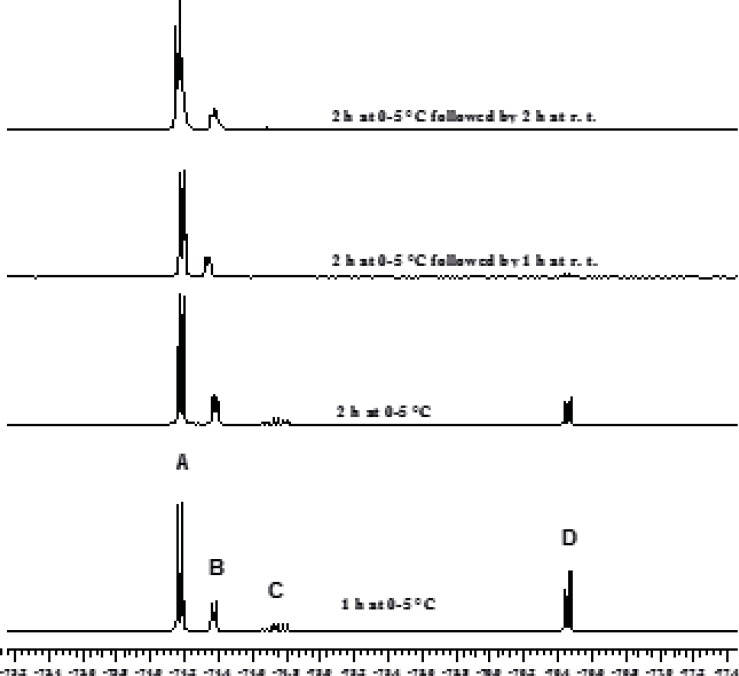
Kinetics of HFIP chloromethylation reaction followed by ^19^F NMR spectroscopy

The chloromethylation reaction of HFIP has been reported to take 24 h and a 1:1:0.3 molar ratio for HFIP, AlCl_3_ and trioxane, respectively, has been used (2, 3). In order to decrease the full conversion time for the first step, the solvent effect was initially investigated. The application of chloroform and dichloromethane resulted in the synthesis of sevochlorane in low yield. Tetrachloroethane led to the formation of bis (HFIP) acetal by-products. Therefore, this solvent has been proposed for the preparation of bis-acetal. Afterwards our attention was shifted to the effect of reagent ratios on the reaction kinetics and reaction yield. It was found out that increasing the amount of AlCl_3_ (only 10 mol%) would sharply accelerates this reaction within the first 2 h and approximately 100% conversion was achieved after 4 h. Increasing the excess value of AlCl_3_ by more than 10% or increasing the amount of trioxane, didn't show further acceleration (Figures 1 and 2). 

**A**: Sevochlorane, **B** and **C**: by-products such as bis(HFIP) acetals and HFIP-methylacetal, **D**: HFIP

The next issue was the high volume of water and acid used for quenching. The addition of 6 N HCl solution to interrupt the reaction, increases the reaction temperature violently, and consequently results in partial product loss by decomposition, evaporation, and polymerization which decreases the first step yield. Considering these disadvantages the aqueous acid and water addition sequence was changed and the bath temperature was increased to dissolve the aluminate salts sufficiently. The HCl gas was conducted to a water vessel and this aqueous HCl, after pH adjustment, was used at the end of the first step (quenching) to dissolve the aluminate salt. The applied changes would certainly reduce environmental pollution and costs.

An important question in relation to the separation of the organic phase in the first step is whether there is any Sevochlorane in the aqueous phase. This question was positively answered by ^19^F NMR analyzing of aqueous phases using 2,2,2-trifluoroethanol, as an internal standard. Thus, all the aqueous phases (used for quenching or washing the organic phase, which were all siphoned off by Bieniarz *et al.*) were recycled and reused in the next batches. 

Halogen-exchange (Halex) fluorination is an important method in preparing fluorinated compounds. Different reagents such as KF, HF, BrF_3_, Bu_4_N^+^F^-^, F_2_, and CsF have been used for this purpose (4-8). Among them, KF, which presents the best ratio between cost and reactivity, is the most popular reagent to perform the "Halex" reaction on a large scale (5). In order to increase the efficiency of KF, severalphase-transfer catalysts such as 18-crown-6, poly (ethylene glycol) (PEG-400) and polar aprotic solvents such as DMSO, DMF and sulfolane have been reported (5-10).Among these, the best result has been obtained when using PEG-400 as the solvent in the case of Sevochlorane (2, 3).

The fluorination of Sevochlorane (SVC) by KF has been reported in the literature and 2.5-7 equivalents of fluoride to SVC have been recommended (2, 3). Trying to reduce the consumed KF and PEG-400 was the main question in this part of our study. To achieve this goal, several reactions using different weight ratios of KF/SVC and PEG/SVC were performed. As shown in Table 2, it is possible to affirm that a decrease of 4 times in the PEG amount (entries 3 and 4) lead to a decrease of less than 1% of sevoflurane conversion and the same is observed when the KF amount is decreased (entries 2 and 3). Accordingly, the amount of KF was decreased to 1.2 mol per each mol of Sevochlorane and the solvent volume was decreased to 3.7 times that of Sevochlorane. An experiment was also carried out using catalytic amount of PEG but the result was not satisfactory because reaching the desired yield required a much longer time (Table 2).

Finally, Sevoflurane was simply isolated from the reaction mixture by the addition of water to the reaction mixture and the organic phase was separated and dried. Analysis of the aqueous phase showed the presence of Sevoflurane. Therefore this phase was used instead of distilled water in subsequent batches. After several batches, tetrachloroethane was added to the accumulated aqueous phases and Sevoflurane was extracted and then the organic phase was distilled to get even more Sevoflurane.

**Table 2:** Influence of amount of solvent and KF on the fluorination of Sevochlorane

**Entry** a	**PEG-400/mL**	**KF/mol L** ^-1^	**Time/h**	**Sevoflurane yield** ** (%)** b	**Unreacted Sevochlorane** ** (%) ** b
**1**	20	0.07	2.5	96.4	3.6
**2**	20	0.04	2.5	95.5	4.5
**3**	20	0.02	2.5	94.5	5.5
**4**	5	0.02	2.5	93.6	6.4
**5**	2.5	0.02	2.5	90.3	9.7
**6**	0.5	0.02	2.5	41.8	58.2
**7**	0.5	0.02	5	51.1	48.9

a All reactions were carried out on a 0.016 mol reaction scale of Sevochlorane in PEG-400 at 95 °C.

b ^19^F NMR assay.

## Conclusion

The two-step synthesis of Sevoflurane has been reinvestigated. Accordingly, the reaction time of the first step was lowered to 4 h and the amount of KF and PEG-400 used in the second step was optimized to minimize the product cost and environmental pollutions. As the solvents were investigated in the first step, tetrachloroethane was found to be a good solvent to direct the chloromethylation step to the bis-HFIP-acetal product. All the aqueous phases, which were all siphoned off by the previous works, were analyzed for the first time and it was found out that these phases contain some product (Sevochlorane and Sevoflurane) that could be recycled. Therefore all these phases together with HCl gas, produced during the first step, were reused in the next batches to decrease the environmental pollutions. Finally all the reactions were monitored by ^19^F NMR for the first time. This process could be easily implemented on larger scales.
